# Low rate of substantial loss of reduction immediately after hardware removal following acromioclavicular joint stabilization using a suspensory fixation system

**DOI:** 10.1007/s00167-022-06978-5

**Published:** 2022-04-22

**Authors:** Marco-Christopher Rupp, Pavel M. Kadantsev, Sebastian Siebenlist, Maximilian Hinz, Matthias J. Feucht, Jonas Pogorzelski, Bastian Scheiderer, Andreas B. Imhoff, Lukas N. Muench, Daniel P. Berthold

**Affiliations:** 1 Department of Orthopaedic Sports Medicine, Technical University of Munich, Ismaninger Str. 22, 81675 Munich, Germany; 2 European Clinic of Sports Traumatology and Orthopaedics (ECSTO), Moscow, Russian Federation; 3 Peoples Friendship University of Russia, Moscow, Russian Federation; 4 Orthopaedic Clinic Paulinenhilfe, Diakonie-Hospital, Stuttgart, Germany; 5 Department of Orthopaedics and Trauma Surgery, Medical Center, Faculty of Medicine, Albert-Ludwigs-University of Freiburg, Freiburg, Germany

**Keywords:** Acromioclavicular joint injury, Acromioclavicular joint instability, AC joint stabilization, Arthroscopically assisted, Hardware removal, Loss of reduction

## Abstract

**Purpose:**

To evaluate immediate loss of reduction in patients undergoing hardware removal after arthroscopically assisted acromioclavicular (AC) joint stabilization using a high-tensile suture tape suspensory fixation system and to identify risk factors associated with immediate loss of reduction.

**Materials and methods:**

Twenty-two consecutive patients with a mean age of 36.4 ± 12.6 years (19–56), who underwent hardware removal 18.2 ± 15.0 months following arthroscopically assisted stabilization surgery using a suspensory fixation system for AC joint injury between 01/2012 and 01/2021 were enrolled in this retrospective monocentric study. The coracoclavicular distance (CCD) as well as the clavicular dislocation/acromial thickness (D/A) ratio were measured on anterior–posterior radiographs prior to hardware removal and immediately postoperatively by two independent raters. Loss of reduction, defined as 10% increase in the CCD, was deemed substantial if the CCD increased 6 mm compared to preoperatively. Constitutional and surgical characteristics were assessed in a subgroup analysis to detect risk factors associated with loss of reduction.

**Results:**

Postoperatively, the CCD significantly increased from 12.6 ± 3.7 mm (4.8–19.0) to 14.5 ± 3.3 mm (8.7–20.6 mm) (*p* < 0.001) while the D/A ratio increased from 0.4 ± 0.3 (− 0.4–0.9) to 0.6 ± 0.3 (1.1–0.1) (*p* = 0.034) compared to preoperatively. In 10 cases (45%), loss of reduction was identified, while a substantial loss of reduction (> 6 mm) was only observed in one patient (4.5%). A shorter time interval between index stabilization surgery and hardware removal significantly corresponded to immediate loss of reduction (11.0 ± 5.6 vs. 30.0 ± 20.8 months; *p* = 0.007), as hardware removal within one year following index stabilization was significantly associated with immediate loss of reduction (*p* = 0.027; relative risk 3.4; odds ratio 11.67).

**Conclusions:**

Substantial loss of reduction after hardware removal of a high-tensile suture tape suspensory fixation system was rare, indicating that the postoperative result of AC stabilization is not categorically at risk when performing this procedure. Even though radiological assessment of the patients showed a statistically significant immediate superior clavicular displacement after this rarely required procedure, with an increased incidence in the first year following stabilization, this may not negatively influence the results of ACJ stabilization in a clinically relevant way.

**Level of evidence:**

IV.

## Introduction

Acromioclavicular (AC) joint injuries account for a significant proportion of shoulder injuries, especially in athletes engaging in contact sports [1, 4, 14, 27, 30, 36]. While current literature supports a non-operative management of low-grade AC joint injuries, Rockwood type IIIB and IV-VI dislocations should generally be treated surgically [1, 13, 29, 31]. With recent literature covering a wide range of open and arthroscopic procedures, a growing body of evidence reports the arthroscopically assisted technique employing suspensory fixation systems to reliably achieve favorable clinical outcomes [10, 13, 22, 29, 37, 38, 41, 43].

Within this technical approach, adequate intraoperative coracoclavicular (CC) reduction is the cornerstone of a successful treatment ensuring long-term stability [17, 22, 26, 29, 33]. While advantages of high-tensile suture tape suspensory techniques include minimal invasiveness without the necessity of hardware removal [5, 25, 40], in rare cases, removal of hardware may be indicated due to mechanical irritation, local pain, or cosmetical reasons. With a high incidence of considerable loss of correction being reported following hardware removal after open AC stabilization using hook plates [18, 32, 40], there is a paucity of evidence investigating the incidence of loss of reduction following hardware removal after arthroscopically assisted stabilization of AC joint separation using high-tensile suture tape suspensory fixation systems.

Thus, the purpose of this retrospective radiographic study was to assess the incidence of immediate postoperative loss of reduction after hardware removal following arthroscopically assisted acromioclavicular joint stabilization using a high-tensile suture tape suspensory fixation system. The secondary objective was to identify risk factors associated with immediate loss of reduction. It was hypothesized that (1) there would be a significant postoperative immediate loss of reduction following hardware removal and that (2) there would be a correlation between immediate loss of reduction and the length of the time interval between primary AC joint injury and index surgery.

## Materials and methods

### Patient selection

This was an Institutional-Review-Board (Technical University of Munich IRB-83/21-S) approved retrospective radiographic outcome study. Patients who underwent hardware removal at the senior author’s institution following arthroscopically assisted stabilization surgery for acute or chronic Rockwood type IIIB, IV, and V injuries (according to the ISAKOS Consensus Statement [2]) using a high-tensile suture tape suspensory fixation system between 01/2012 and 01/2021 were screened for eligibility. Only patients treated with primary CC stabilization surgery using a high-tensile suture tape suspensory fixation system (Arthrex, Naples, FL, USA) were included. Patients were indicated for hardware removal due to cosmetical reasons, mechanical irritation or pain caused by the cranial button-suture construct, refractory to conservative treatment. Furthermore, preoperative radiographs prior to hardware removal and within a maximum of 2 days postoperatively as well as comprehensive medical records were required for inclusion. Patients were excluded if they underwent CC stabilization using a technique other than a high-tensile suture tape suspensory fixation system; had concomitant fractures of the lateral clavicle or coracoid; underwent CC implant removal due to infection; underwent incomplete hardware removal; or were considered revision cases.

### Radiographic evaluation

**Fig. 1:**
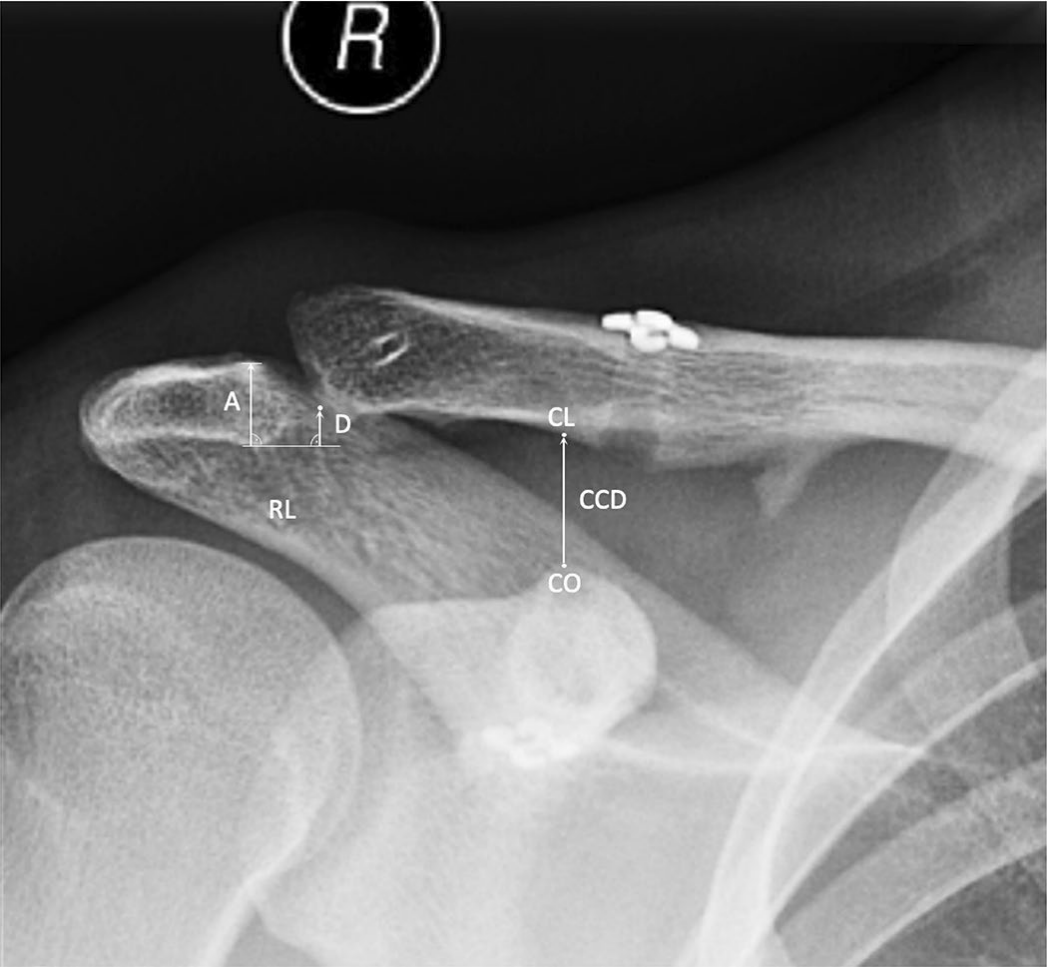
Measurement protocol. “CO”, tip of the coracoid; “CL”, inferior cortex of the clavicle; distance “CCD”, coracoclavicular distance measured between CO and CL; distance “A”, acromial thickness measured as the distance between the superior and inferior margin of the acromion; line “RL”, reference line at the inferior acromial cortex placed perpendicularly to A; distance “D”, distance between RL and the lowest and most lateral point on the clavicle measured perpendicularly to RL

Radiographic analysis (Fig. 1) was performed on unweighted anterior–posterior view radiographs, as validated in previous studies [21, 45], two times at an interval of one month by the main observer (MCR) for intrarater reliability and additionally by a second observer (PK) for inter-rater reliability. Each patient served as its own control for further radiographic assessment. Measurements were performed employing validated protocols [3, 16, 42]: the coracoclavicular distance (CCD) was measured as the distance between the tip of the coracoid and the inferior cortex of the clavicle as previously described [3, 42]. To quantify a potential clavicular displacement in relation to the acromion, the displacement/acromial thickness (D/A) ratio was measured as previously proposed [16]: a reference line (RL) was placed at the inferior margin of the acromion. Distance A was measured between the superior and inferior margin of the acromion. Distance D was defined as the distance between RL and the lowest and most lateral point on the clavicle, measured perpendicularly to the RL, with negative values indicating an overcorrection at index surgery. Consecutively, the ratio between clavicular displacement in relation to the acromial thickness, the D/A ratio, was calculated [16]. Radiographic measurements were performed using a digital ruler (accuracy 0.1 mm) via a DICOM medical imaging viewer using the picture archiving and communication system (PACS). Loss of reduction after hardware removal was defined as an 10% increase in the CCD compared to preoperatively (prior to hardware removal), while loss of reduction was deemed substantial if the CCD increased 6 mm postoperatively compared to preoperatively, as previously proposed [8, 34, 39]. Horizontal instability was not assessed in this study, as stress radiographs in crossbody adduction views were not routinely performed immediately following surgery at the senior author’s institution.

### Surgical technique

Index stabilization was performed either via isolated CC stabilization using a high-tensile suture tape suspensory fixation system (Arthrex, Naples, FL, USA) or combined CC stabilization and AC cerclage [44]. Isolated CC ligament reconstruction was performed under fluoroscopic control via an arthroscopically assisted technique employing a suspensory fixation system composed of two high-strength suture tapes (FiberTape, Arthrex, Naples, FL, USA), while fixation was performed using two endobuttons (DogBone, Arthrex, Naples, FL, USA). AC cerclage was performed open using an 1.5-mm polydioxanone cord either in a “box” or “figure of 8” technique [44]. The use of the additional AC cerclage did not follow any specific protocol. When tendon augmentation was indicated, a gracilis tendon autograft was shutteled transclavicular and transcoracoidal along with the FiberTape (Arthrex, Naples, FL, USA) and then passed lateral to the coracoid to the top of the clavicle to complete the loop, where it was consecutively knotted and secured via an absorbable suture below the endobutton fixation.

The hardware removal procedure was performed following examination under anesthesia for AC joint instability. After diagnostic arthroscopy through a standard posterior viewing portal, the arch and base of the coracoid were prepared with an electrothermal ablation device through an anterolateral working portal to visualize the endobutton caudal of the coracoid. Subsequently, the clavicular endobutton was removed via mini-open technique. Consecutively, the subcoracoid button was grasped using an arthroscopic grasper (KingFisher, Arthrex, Naples, FL, USA) and the button-suture construct was carefully retrieved through the anterolateral working portal. After a final assessment of the subcoracoid space and the glenohumeral joint for remaining suture material, incisions were then closed in a sterile fashion.

### Postoperative rehabilitation

After initial limitation of the patient’s passive range of motion, free active ROM was permitted after 2 weeks and return to overhead activity and return to full-contact sports were allowed after 6 weeks postoperatively.

### Subgroup analysis

The association between risk factors including preoperative demographic and surgical characteristics of the patient cohort and postoperative loss of reduction were assessed via subgroup analysis. According to the radiographic assessment, patients were either assigned to the group with or without loss of reduction. The size of the study population statistically limited the number of risk factors to be evaluated, since repeatedly testing an excessive number of factors on a single dataset predisposes for the occurrence of type 1 (false-positive) errors. Thus, the following preoperative factors a priori for assessment of the second hypothesis in this study were selected: constitutional factors (BMI, age), preoperative clinical characteristics (time between AC joint injury and index stabilization surgery, time between index stabilization surgery and hardware removal,) and surgical details (CCD and D/A ratio prior to hardware removal). To avoid underpowering and to reduce the risk for a type II error, only comparisons with group size  n>10 were considered for subgroup analysis. Thus, factors such as sex, overhead activity in sports or work, Rockwood type prior to index surgery, indication for hardware removal and concomitant AC cerclage were excluded from the subgroup analysis.

### Statistical analysis

Descriptive statistics including mean and standard deviation for continuous variables as well as frequency and proportion for categorical variables were calculated to characterize the study collective. The distribution of continuous variables in the study collective was categorized via Shapiro–Wilk test and did not confirm to a normal distribution. The Wilcoxon sign rank test (non-parametric analogue to the dependent *t* test) was used to compare a pre-to postoperative change in the CCD as well as D/A ratio. Two-way random interclass correlation coefficient (ICC) were used to assess the reliability of the measurements of CCD. ICC values were calculated for consistency of agreement. ICC values were graded as following: < 0.4 poor reliability, 0.4–0.75 moderate reliability, and > 0.75 excellent reliability.

A *p* value of less than 0.05 was set to be statistically significant. For the subgroup analysis, categorical variables were compared performing the binary Fisher’s exact test or the Chi-square test, where statistically appropriate, while continuous variables were compared employing the non-parametric Mann Whitney *U* test. All analyses were performed using SPSS software version 26.0 (IBM-SPSS, New York, USA). A total sample size of 21 subjects to detect a difference of 1 mm of the primary endpoint measurement, the CCD, with an assumed standard deviation of 1.5 mm at a calculated effect size of 0.66 in order to achieve a statistical power of 0.8 was determined in an a-priori power analysis, performed with G × Power (Erdfelder, Faul, Buchner, Lang, HHU Düsseldorf, Düsseldorf, Germany) [12].

## Results

**Fig. 2:**
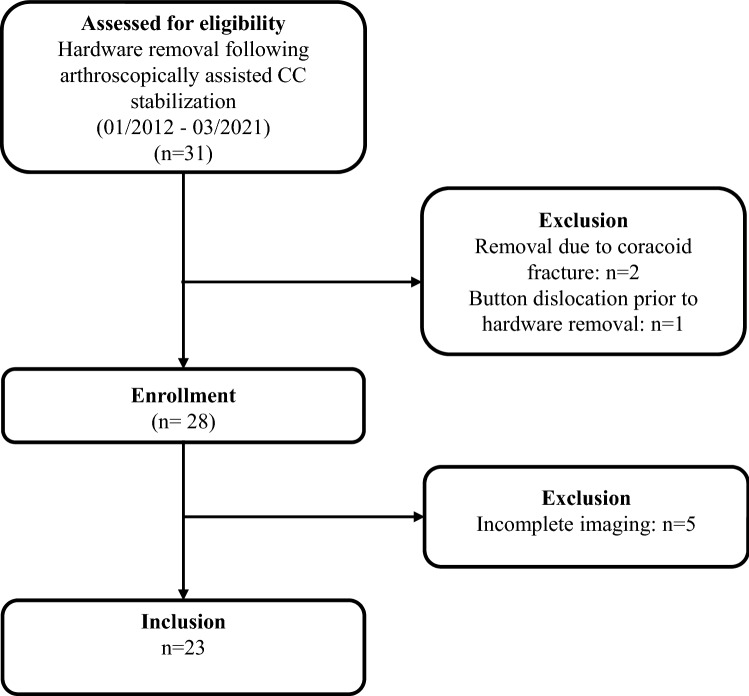
Flow chart visualizing the patient population for this study after accounting for inclusion and exclusion criteria. *CC* coracoclavicular

At the authors’ institution, 31 patients who underwent hardware removal following arthroscopically assisted CC stabilization with suspensory fixation system for AC joint injury between 01/2012 and 01/2021 were identified through review of the institutional database, accounting for less than 5% of the CC stabilization procedures performed during inclusion period. Of those, 9 patients were excluded for either having concomitant fractures of the coracoid, presenting with infection or coracoid button dislocation, or incomplete radiographic imaging. Thus, the final study population comprised 22 patients (18 men, 4 women, mean age 36.4 ± 12.6 years, 19–56) (Fig. 2, Table 1). Patient demographics are demonstrated in Table 1.

**Table 1:** Description of study group

Variable	Total study group
Patients	22
Sex	
Male	18 (82%)
Female	4 (18%)
Age (years)^a^	36.4 ± 11.6 (19–56)
BMI (kg)	23.7 ± 2.6 (19.3–27.1)
Smoking	15 (68%)
Alcohol	0 (0%)
Comorbidities	0 (0%)
Laterality	
Right	11 (50%)
Left	11 (50%)
Etiology^b^	
Acute	19 (86%)
Chronic	3 (14%)
Rockwood grade	
IIIB	3 (14%)
IV	7 (32%)
V	12 (54%)
Suspensory systems	
One	20 (91%)
Two	2 (9%)
Concomitant procedures at index surgery	
Tendon augmentation	2 (9%)
AC-cerclage	12 (55%)
Biceps tenodesis	1 (5%)
SLAP repair	1 (5%)
Indication for hardware removal^c^	
Local pain^d^	10 (45%)
Mechanical irritation	16 (73%)
Time to hardware removal^e^	
(months)	18.2 ± 15.0 (6–73)
Hardware removal procedure	
One suspensory system	20 (91%)
Two suspensory systems	2 (9%)
Removal of AC-cerclage	1 (5%)
AC joint denervation	1 (5%)

Continuous variables are presented as mean ± standard deviation (range); Categorical variables are presented as count and percentage

*BMI* body-mass-index, *SLAP* superior labrum anterior to posterior

^a^Age at surgery

^b^Definition according to the ISAKOS Consensus Statement; acute < 3 weeks after trauma; chronic > 3 weeks after trauma

^c^Total number exceeds 22 (total study group), as certain patients were indicated for hardware removal for more than one reason

^d^Local pain over the titanium button on the clavicle

^e^Time between index CC stabilization surgery and hardware removal

### Radiographic analysis

**Fig. 3:**
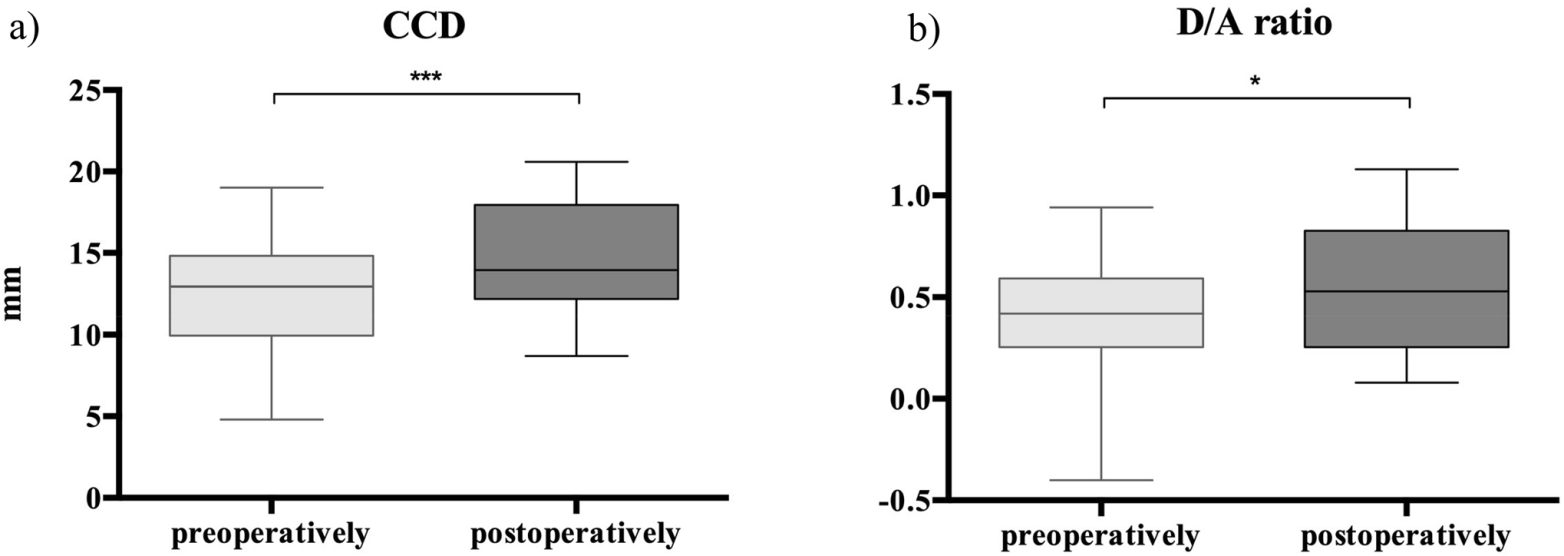
Boxplot graph visualizing pre- and postoperative outcomes of the radiological analysis for **a** coracoclavicular distance (CCD) and **b** dislocation/acromial thickness (D/A) ratio measurement for the hardware removal procedure. Boxes: median ± Q1 and Q3, whiskers: min. and max value of each data set, *p < 0.05, **p < 0.001, ***p < 0.001

When compared to preoperatively, postoperative CCD significantly increased from 12.6 ± 3.7 (4.8–19.0) to 14.5 ± 3.3 (8.7–20.6) (*p* < 0.001). Furthermore, postoperative superior clavicular displacement, as quantified by the D/A ratio, increased from 0.4 ± 0.3 mm (− 0.4–0.9 mm) to 0.6 ± 0.3 mm (0.1–1.1 mm) (*p* = 0.034) compared to preoperatively (Fig. 3). As such, the incidence of loss of reduction was 45% (10 of 22 cases), while the incidence of a substantial loss of reduction compared to preoperatively was 5% (1 of 22 cases).

### Interrater reliability

Intrarater reliability were calculated for CCD and D/A ratio and was found to be excellent for both CCD (ICC = 0.94; CI 0.89–0.97) and D/A ratio (ICC = 0.94; CI 0.88–0.97). Similarly, interrater reliability was excellent for both CCD (ICC = 0.97; CI 0.95–0.99) and D/A ratio (ICC = 0.93; CI 0.86–0.97).

### Risk factor analysis

Analyzing the collective for risk factors associated with postoperative loss of reduction, the time interval between index stabilization surgery and consecutive hardware removal was significantly shorter in cases with loss of reduction (11.0 ± 5.6 months; 6.0–25.0) compared to cases not subject to loss of reduction (30.0 ± 20.8 months; 8.0–73.0) (*p* = 0.007). Hardware removal before a minimum of 1 year following index stabilization significantly predisposed for the incidence of a loss of correction (*p* = 0.027; relative risk 3.4; odds ratio 11.67). The demographic factors (BMI, age), the factors specific for the index procedure (time between trauma and index surgery) and the radiological factors prior to hardware removal procedure (preoperative CCD or D/A ratio) that were sub-analyzed were not found to be significantly associated with a postoperative loss of reduction (Table 2).

**Table 2:** Subgroup analysis

Variable	Loss of reduction		*p* value
	No	Yes	
Cases	11	11	
Age^a^	33.3 ± 8.4 (22–49)	40.1 ± 14.0 (19–56)	n.s
BMI	23.2 ± 2.4 (19.9–26.8)	23.5 ± 2.9 (19.3–27.1)	n.s
Time to index surgery^b^ (days)	47.0 ± 119.0 (3–365)	30.0 ± 60.0 (4–200)	n.s
Time to hardware removal^c^ (months)	30.0 ± 20.8 (8–73)	11.0 ± 5.6 (6–25)	0.007*
Preoperative CCD (in mm)	13.6 ± 3.2 (8.5–19.0)	11.4 ± 4.2 (4.8–17.4)	n.s
Preoperative D/A ratio	0.5 ± 0.2 (0.2–0.9)	0.3 ± 0.3 (− 0.4–0.6)	n.s

Continuous variables are presented as mean ± standard deviation (range); Categorical variables are presented as count and percentage

*BMI* body-mass-index, *AC* acromioclavicular

^a^Age at surgery

^b^Time between trauma and index CC stabilization surgery

^c^Time between index CC stabilization surgery and hardware removal

*denotes statistical significance with a p-value <0.05

## Discussion

The most important finding of the study was that substantial loss of reduction after hardware removal of a high-tensile suture tape suspensory fixation system was rare, indicating that the postoperative result of AC stabilization is not categorically at risk when performing this procedure. Even though radiological assessment of the patients showed a statistically significant immediate superior clavicular displacement, this may be negligible and not of clinical relevance. Interestingly, time between the index stabilization and hardware removal was associated with the incidence of an immediate loss of reduction. This may be explained by the fact that these patients may present with an elongated CC-complex which failed to keep the AC joint reduced without additional suture tapes.

In reference to current orthopedic literature, the results of this study underscore the previously propagated capability of arthroscopically assisted stabilization surgery using a suspensory fixation system to provide a reliable coracoclavicular reduction [17, 20, 22, 28, 29, 41], with a mean postoperative CCD of 12.6 ± 3.7 mm at a mean follow-up of 18.2 ± 15.0 months. Moreover, findings of the current study fall within range of these previous studies reporting a postoperative CCD of 9.2–13.9 mm using comparable techniques [20, 22, 28, 41]. As quantified by the low D/A ratio indicating anatomical reduction, a satisfactory coracoclavicular reduction was achieved across the entire collective. Further, the finding of non-substantial loss of reduction prior to hardware removal, as indicated by a D/A ratio of 0.4 ± 0.3 in this patient cohort, is well reflected by previously published radiographic outcome data, documenting an increase in CCD of 1.1–2.8 mm when compared to the contralateral side [17, 23, 38].

A significant advantage of arthroscopically assisted surgery with suspensory fixation systems compared to rivaling technical options such as hook plate stabilization is that subsequent removal of hardware is not mandatory, as indications including mechanical irritation and pain are rarely encountered [5, 40]. Thus, there is a paucity of evidence on the rarely required hardware removal procedure following suspensory fixation in the setting of AC joint stabilization. During the inclusion period, less than 5% of all arthroscopically assisted CC stabilizations at the author’s institution underwent consecutive hardware removal for local pain and mechanical irritation over the cranial fixation button—symptoms that, while not equaling clinical failure of AC joint stabilization, may represent a substantial subjective burden to the individual patient. In addition, the relatively healthy and athletic patient population included in this study represents the typical collective affected by AC joint injury, with a slight overrepresentation of female sex (18% women) and relatively low BMI of the study population, indicating a potential aesthetic component in pursuing hardware removal [15, 29, 44].

While—accounting for the high ICC values of the measurements — the increased CCD of 2.0 ± 1.9 mm after hardware removal, which is 20-fold larger that applied measurement accuracy of 0.1 mm, is quantifiable, it seems to be marginal when compared to hardware removal after hook plate fixation. CCD increases between 3 and 13 mm [11, 18, 25] and a loss of reduction of more than 2 mm have been reported in 68% of the patients [19] following hardware removal after hook plate fixation. Acknowledging that a substantial loss of reduction was rare (< 5%), removal of the suspensory fixation system does not categorically jeopardize the postoperative result of AC joint stabilization. While previous outcome studies could not determine a correlation between a moderate loss of reduction of 3–4 mm and clinical outcomes [6, 7, 35], the clinical relevance of a significant CCD increase following hardware removal yet remains to be investigated in a prospective approach.

Acknowledging the finding of an increased incidence of superior clavicular displacement following hardware removal performed within one year after the index stabilization, clinically actionable insight should however be derived cautionary. Hypothesizing retrospectively, a disruption of an ongoing biological healing process in terms of collagen remodeling from type III to biomechanically superior type I collagen during the first year might be causative [9, 24]. However, an a priori failure of biological CC healing, resulting in an increased mechanical shear stress and consequentially increased mobility of the implant construct may translate to increased pain for the patient and thus incentivize affected patients to seek implant removal.

The analysis of this investigation must be interpreted within the context of the study's limitations. First, as stress radiographs in crossbody adduction views were not routinely performed following surgery to consolidate the postoperative result, no statement can be made on the postoperative incidence of horizontal instability. Second, as the purpose of this study was to evaluate CCD immediately after hardware removal, excluding confounding by secondary dynamical stress during rehabilitation, the analysis is limited to early radiological outcomes, with follow-up investigations being warranted. Third, as incidence of loss of reduction was elected as the primary endpoint of this retrospective radiographic analysis, reporting of clinical outcome exceeded the scope of this study. Fourth, while comparable to previous radiologic investigations of the AC joint [3, 42] the sample size of this study was relatively small due to the rarity of the condition and the strict inclusion criteria, thus potentially predisposing for the incidence of a statistical type II error as well as statistical type I error, especially in the subgroup analyses. Ultimately, with five patients lost due to insufficient imaging, the study inherits the associated biases of a retrospective design.

The findings of this study may be of clinical relevance to the surgeon confronted with patients asking for hardware removal of the high-tensile suture tape suspensory fixation system after AC joint stabilization. This study provides evidence that performing hardware removal does not categorically jeopardize the postoperative radiological result of AC stabilization, as immediate superior clavicular displacement is only minor and a substantial loss of reduction is rare.

## Conclusion

Substantial loss of reduction after hardware removal of a high-tensile suture tape suspensory fixation system was rare, indicating that the postoperative result of AC stabilization is not categorically at risk when performing this procedure. Even though radiological assessment of the patients showed a statistically significant immediate superior clavicular displacement after this rarely required procedure, with an increased incidence in the first year following stabilization, this may not negatively influence the results of ACJ stabilization in a clinically relevant way.

## Abbreviations

AC: Acromioclavicular
BMI: Body mass index
CCD: Coracoclavicular distance
CL: Clavicle
CC: Coracoclavicular
CO: Coracoid
D/A ratio: Distance/acromial thickness ratio
RL: Reference line
